# Pneumopathie postoperatoire à association Haemophilus Influenzae et Neisseria meningitidis chez un enfant diabetique

**DOI:** 10.11604/pamj.2016.25.84.7290

**Published:** 2016-10-17

**Authors:** Hicham Chemsi, Mohamed Frikh, Abdelhay Lemnouer, Bouchra Belfkih, Yassine Sekhsokh, Maryama Chadli, Mustapha Elouennass

**Affiliations:** 1 Laboratoire de Bactériologie de l’Hôpital Militaire d’Instruction Mohamed V de Rabat, Faculté de Médecine et de Pharmacie de Rabat, Université Mohammed V Souissi, Rabat, Maroc; 2 Equipe de Recherche Epidémiologie et Résistance Bactérienne, Faculté de Médecine et de Pharmacie de Rabat, Université Mohammed V-Souissi, Rabat, Maroc; 3 Laboratoire de Recherche et de Biosécurité de l’Hôpital Militaire d’Instruction Mohamed V de Rabat, Faculté de Médecine et de Pharmacie de Rabat, Université Mohammed V-Souissi, Rabat, Maroc

**Keywords:** Neisseria meningitidis, haemophilus influenzae, association, pneumopathie, Neisseria meningitidis, haemophilus influenzae, association, pneumonitis

## Abstract

*Haemophilus influenzae* est un hôte saprophyte du rhinopharynx chez près des deux tiers des enfants et les adultes. *Neisseria meningitidis* est une bactérie strictement humaine qui vit dans le rhinopharynx, pouvant provoquer une rhinopharyngite bénigne ou un état de portage asymptomatique. Nous rapportons le cas d'une pneumopathie postopératoire à association *Haemophilus influenzae* et *Neisseria meningitidis* chez un enfant diabétique. Patient âgé de 3 ans, diabétique admis au service de chirurgie cardio-vasculaire pour prise en charge chirurgicale tardive. L'évolution postopératoire a été marquée par une aggravation de l'état respiratoire, devenu encombré avec des secrétions abondantes nécessitant une hospitalisation en réanimation. Un bilan infectieux a été réalisé, notamment un prélèvement distal protégé qui a révélé une association de *Neisseria meningitides* et *Haemophilus influenzae*. A travers ce cas, nous discutons les associations bactériennes dans certaines situations à risque. Chacune de ces deux espèces est responsable d'infections diverses. Cependant l'association au même site est rare.

## Abstract (en)

Haemophilus influenzae is a saprophyte that colonizes the nasopharynx in nearly two thirds of children and adults. Neisseria meningitidis is a strict human bacterium which lives in the nasopharynx. It can cause benign nasopharyngitis or asymptomatic colonization. We report the case of a diabetic child with postoperative pneumonia associated with Haemophilus influenzae and Neisseria meningitidis. A 3-year old diabetic patient admitted to the Department of Cardiac Surgery due to delayed surgical treatment. The postoperative course was marked by a worsening of respiratory status caused by abundant secretions requiring patient admission to the intensive care unit. An assessment for the detection of infections was performed, including protected distal sampling which revealed the association of Neisseria meningitidis with Haemophilus influenzae. This case study allowed us to highlight bacterial associations in certain high-risk situations. Each of these two species is responsible for various infections. However their presence in the same infected site is rare.

## Introduction

*Neisseria meningitidis* est une bactérie classique en pathologie infectieuse, responsable d'infections méningées. Les formes cliniques les plus rencontrées sont la méningite cérébro-spinale et la méningococcémie aigue. Les formes atypiques sont également articulaires, cutanées, cardiaques, uro-génitales, abdominales, oculaires et pulmonaires. *Haemophilus influenzae*, bactérie potentiellement pathogène, est un hôte fréquent du rhinopharynx. L'infection est le résultat d'une colonisation. La connaissance des paramètres de la colonisation et de l'infection permet de déterminer les facteurs de risque, dont le jeune âge. Nous rapportons le cas rare d'une pneumopathie postopératoire à l'association *Haemophilus influenzae* et *Neisseria meningitidis* chez un enfant diabétique.

## Patient et observation

Patient âgé de 3 ans, diabétique admis au service de chirurgie cardio-vasculaire pour prise en charge chirurgicale tardive, d'une communication inter-ventriculaire péri-membraneuse avec retard pondéral. L'échographie cardiaque transthoracique avait objectivé une CIV PM, de 14 mm, en hypertension artérielle pulmonaire. L'évolution postopératoire a été marquée par une aggravation de l'état respiratoire, devenu encombré avec des secrétions abondantes nécessitant une hospitalisation en réanimation. Un bilan infectieux a été réalisé, un prélèvement distal protégé qui était d'un aspect purulent à l'examen macroscopique. L'examen microscopique de la parcelle purulente recueillie comportait d'abord une étude cytologique pour apprécier le nombre de cellules épithéliales (indice de la contamination oropharyngée), la richesse en polynucléaires, l'abondance des cellules bronchiques. D'autre part l'examen bactériologique retenait les germes présents dans ou autour des polynucléaires neutrophiles. Leur abondance (cocci à Gram négatif) était notée, ainsi que leur localisation intra leucocytaire. Les cultures ont été effectuées, incubées et observées à 24 heures et 48 heures. La culture était riche et significative associant deux espèces *H. influenzae* et *N. meningitidis*. La richesse de la culture pour chaque espèce présumée pathogène a été notée. L'antibiogramme qui a été réalisé, indiquait une résistance d'*H.influenzae* aux aminopenicillines et *N.meningitidis* qui était multi sensible aux antibiotiques. Le patient a été traité par une Céphalosporine de 3^ème^ génération avec une bonne évolution.

## Discussion

Les localisations extraméningées isolées du méningocoque sont rares et habituellement rapportées sous la forme de cas cliniques isolés. Les localisations extraméningées les plus fréquemment décrites sont les bactériémies (5 à 20% des cas) et les pneumopathies (5 à 15%) [1, 2]. Les pneumopathies à *N. meningitidis*touchent une population âgée de plus de 40 ans. Les autres localisations sont rares et variées. Elles peuvent être oculaires (panophtalmie, endophtalmie, conjonctivite purulente) [3, 4], cardiaques (endocardite, péricardite, myocardite, troubles de la conduction) [5], rhumatologiques (arthrite septique ou réactionnelle, ostéomyélite) [6], urogénitales et digestives (urétrite, péritonite, ascite) [7]. Le diagnostic de pneumopathie à méningocoque est sujet à controverse car les crachats peuvent être contaminés par un portage pharyngé simple. Par ailleurs, le méningocoque est souvent associé à un autre germe [8]. La survenue d'une infection à méningocoque est favorisée par l'existence d'une immunodépression (infection par le VIH, personne âgée, corticothérapie, hémopathie, maladie de système [9, 10]). Les personnes souffrant d'un déficit de l'immunité humorale médiée par le complément ont plus de risque de développer une infection à méningocoque. Le risque de méningococcémie est plus élevé chez les patients exposés au tabac et lors d'infections virales pharyngées, probablement par altération de la barrière muqueuse du nasopharynx [11]. *Haemophilus influenzae* est une bactérie commensale, dont il existe de nombreuses variétés, la majorité est non capsulée et pouvant doter occasionnellement des infections. Dans les infections systémiques ou elle est en cause, la bactérie est pratiquement toujours capsulée. Le rôle des pili (= fimbriae) est moins clair pour *H. influenzae* que pour le méningocoque. En dehors des pilis, il existe des adhésines facilitant l'attachement à la muqueuse, interagissant avec des récepteurs.

Les souches piliées seraient plus invasives [12]. Les prélèvements protégés de type ponction transtrachéale, aspiration bronchique, brossage bronchique ou aspiration de liquide pleural sont plus intéressants que le classique crachat et leur positivité aide à faire le diagnostic d'une infection broncho-pulmonaire à *N. meningitidis*et *H.influenzae* [13–15]. Le méningocoque est, une fois sur deux associé à d'autres bactéries pathogènes tels que le pneumocoque, *H.influenzae*, le staphylocoque doré, le bacille de Koch [16]. Le pronostic des infections extraméningées à méningocoque est différent chez l'adulte et chez l'enfant. Chez l'adulte, il existe constamment une immunodépression associée, les localisations septiques sont diverses, la présentation clinique souvent déroutante, le diagnostic retardé et le pronostic sévère, l'infection est recherché systématiquement par une ponction lombaire et un prélèvement de gorge réalisé d'emblée. À l'inverse, chez l'enfant, la présentation clinique est plus homogène que chez l'adulte. La fièvre est le motif principal d'hospitalisation.la clinique est plus stéréotypé, l'evolution est plus rapidement favorable, avec une antibiothérapie de courte durée. Selon une étude, d'après le suivi de 468 enfants pendant 18 mois [17], la colonisation par *H. influenzae* s'établit graduellement. Elle passe de 5% à 1-3 mois à 24% à 18 mois, alors que la colonisation par pneumocoque est plus rapide, atteignant un maximum à 4-7 mois. La fréquence de la colonisation par *H. influenzae* augmente en fonction de la fratrie et du séjour en crèche. Chez l'adulte, la fréquence de la colonisation diminue modérément, quatre adultes sur dix contre huit enfants sur dix [18].

## Conclusion

Les infections à l'association du méningocoque et *Haemophilus* ne sont pas exclusivement représentées par la méningite ou la méningococcémie. On retrouve aussi des formes broncho-pulmonaires. Elles passent souvent inaperçues. Les manifestations extraméningées sont beaucoup plus rares. Un intérêt tout particulier doit être porté aux manifestations broncho-pulmonaires jusqu'à nos jours négligées. A travers ce cas, nous avons rapporté les associations bactériennes dans certaines situations à risque. Chacune de ces deux espèces est responsable d'infections diverses. Cependant l'association au même site est rare.
